# Supplementary material for: A lignin-derived material improves plant nutrient bioavailability and growth through its metal chelating capacity
Source: Nat Commun. 2023 Aug 11;14:4866. doi: 10.1038/s41467-023-40497-2 (PMC10421960; doi:10.1038/s41467-023-40497-2)
Supplement: Supplementary file 1 — Supplementary Information [file 41467_2023_40497_MOESM1_ESM.pdf]

**Supplementary Information**

# A lignin-derived material improves plant nutrient bioavailability and growth through its metal chelating capacity

Qiang Liu[1,2], Tsubasa Kawai[2], Yoshiaki Inukai[3], Dan Aoki[2], Zhihang Feng[1], Yihui Xiao[1], Kazuhiko Fukushima[2], Xianyong Lin[1], Weiming Shi[4,5], Wolfgang Busch[6], Yasuyuki Matsushita[*2,7] Baohai Li[*1]

[*]**Corresponding authors**
Correspondence to Yasuyuki Matsushita, fx7789@go.tuat.ac.jp; Baohai Li, bhli@zju.edu.cn.

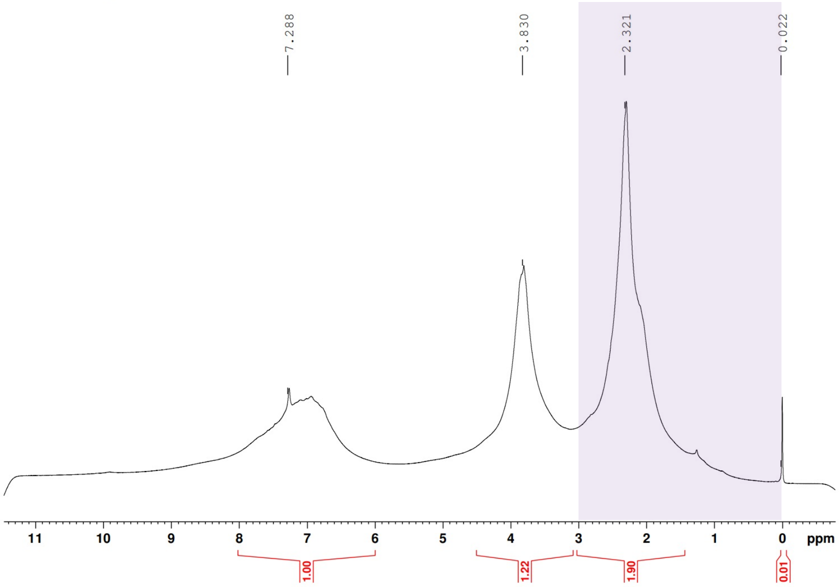


**Supplementary Fig. 1 | $^1$H-NMR spectra of acetylated HSAL.** Quantification of the phenolic hydroxyls (2.2 ppm to 2.6 ppm) was done by peak integration in comparison with dibromomethane internal standard peaks (The spectra were recorded in a deuteriochloroform solvent at 25 °C using a Bruker AVANCE 400 MHz NMR spectrometer). The purple area indicates 1H -NMR spectra of acetylated HSAL at 1 ~ 3 ppm (shown in Fig. 1b).

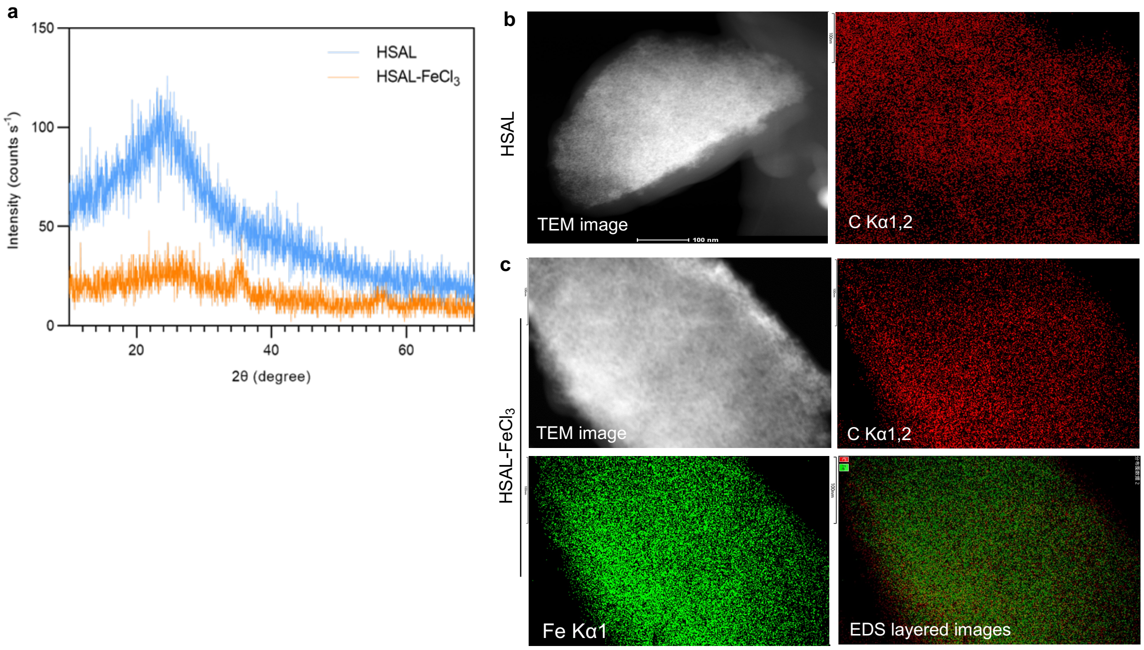


**Supplementary Fig. 2 | XRPD and TEM-EDS analysis of HSAL and HSAL-$FeCl_3$ complexes.** (**a**) Overlap of XRPD plots of HSAL powder and HSAL-$FeCl_3$ complexes. (**b**) HAADF-STEM and TEM-EDS analysis and carbon distribution on the surface of HSAL powder. (**c**) HAADF-STEM and TEM-EDS analysis and carbon-iron distribution on the surface of HSAL-$FeCl_3$ powder.

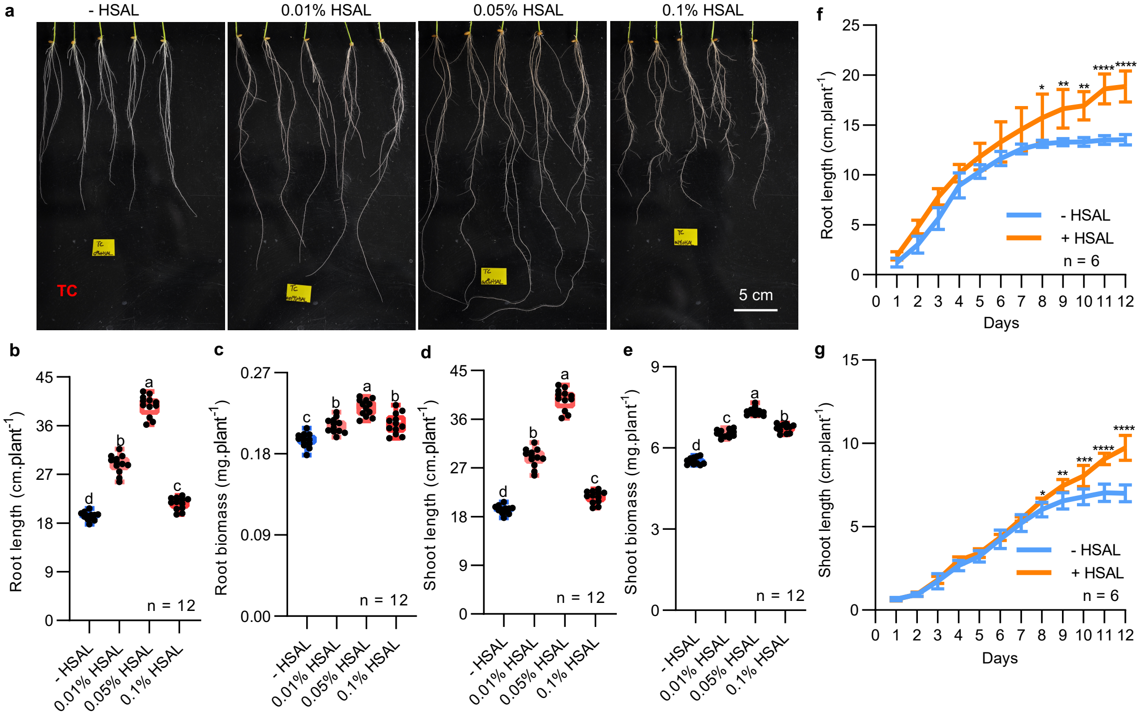


**Supplementary Fig. 3 | The significant promotion of HSAL additive on rice (Taichung 65) growth. a,** The representative images showed the root length of rice seedlings treated with different concentrations of HSAL for 14 days. **b, c,** Quantification of the primary root length (**b**) and total root biomass (**b**) in (**a**). **d, e,** Quantification of the shoot length (**d**) and total shoot biomass (**e**) in (**a**). Boxes represent the 25$^{th}$–75$^{th}$ percentile range, white lines represent medians and whiskers show the minimum–maximum range in (**b, c, d, e**). Different letters indicated statistically significant differences based on one-way ANOVA and Tukey's *HSD* test analysis ($p < 0.05$). **f, g,** Growth curve on the primary root length (**f**) and shoot length (**g**) of *Taichung 65* with 0.05% HSAL. The data indicated the mean and standard deviation (error bars). Statistical difference between the control and 0.05% HSAL was analyzed with Student's *t*-test in (**f, g**). Different number of asterisk (*) indicates *p*-value at the level of 0.05, 0.01, 0.001 and 0.0001, respectively. The number of seedlings (n) shown in (**b, c, d, e, f, g**).

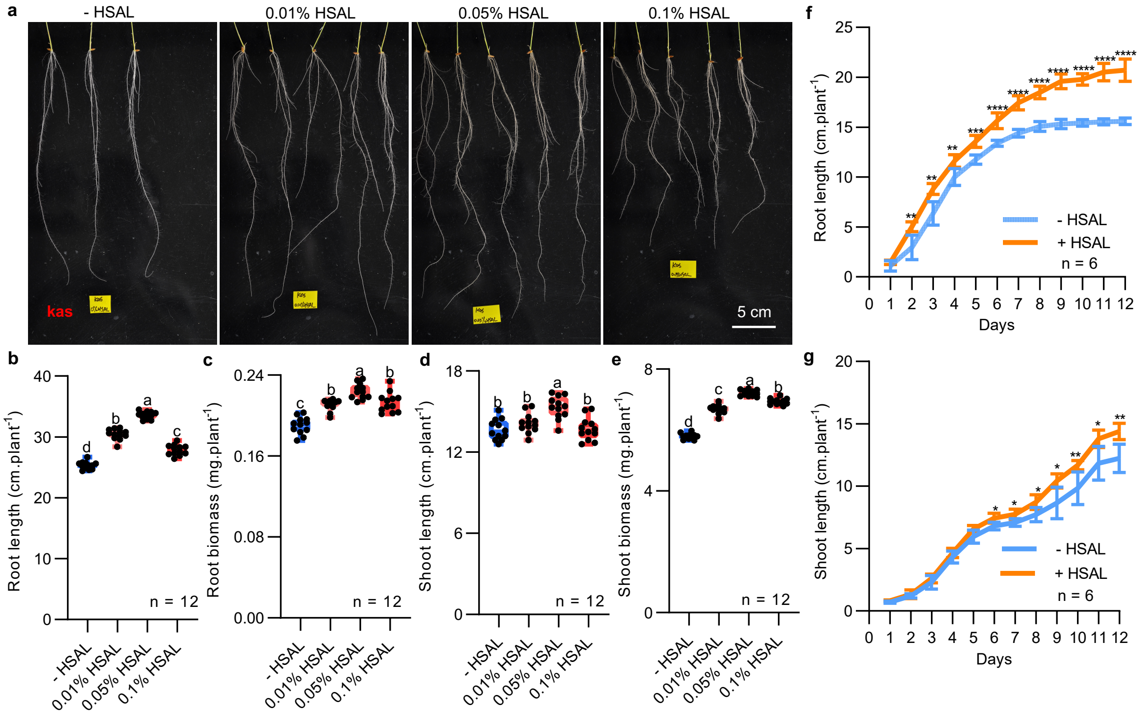


**Supplementary Fig. 4 | The significant promotion of HSAL additive on rice *Kasalath* (*Kas*) growth. a,** The representative images showed the root length of *Kas* rice seedlings treated with different concentrations of HSAL for 14 days. **b, c,** Quantification of the primary root length (**b**) and total root biomass (**c**) in (**a**). **d, e,** Quantification of the shoot length (**d**) and total shoot biomass (**e**) in (**a**). Boxes represent the 25th–75th percentile range, white lines represent medians and whiskers show the minimum–maximum range in (**b, c, d, e**). Different letters indicated statistically significant differences based on one way ANOVA and Tukey's *HSD* test analysis ($p < 0.05$). **f, g,** Growth curve on the primary root length (f) and shoot length (**g**) of *Kas* rice seedlings treated with the control and 0.05% HSAL. The data indicated the mean and standard deviation (error bars). Statistical difference between the control and 0.05% HSAL was analyzed with Student's *t*-test in (**f, g**). Different number of asterisk (*) indicates *p*-value at the level of 0.05, 0.01, 0.001 and 0.0001, respectively. The number of seedlings (n) shown in (**b, c, d, e, f, g**).

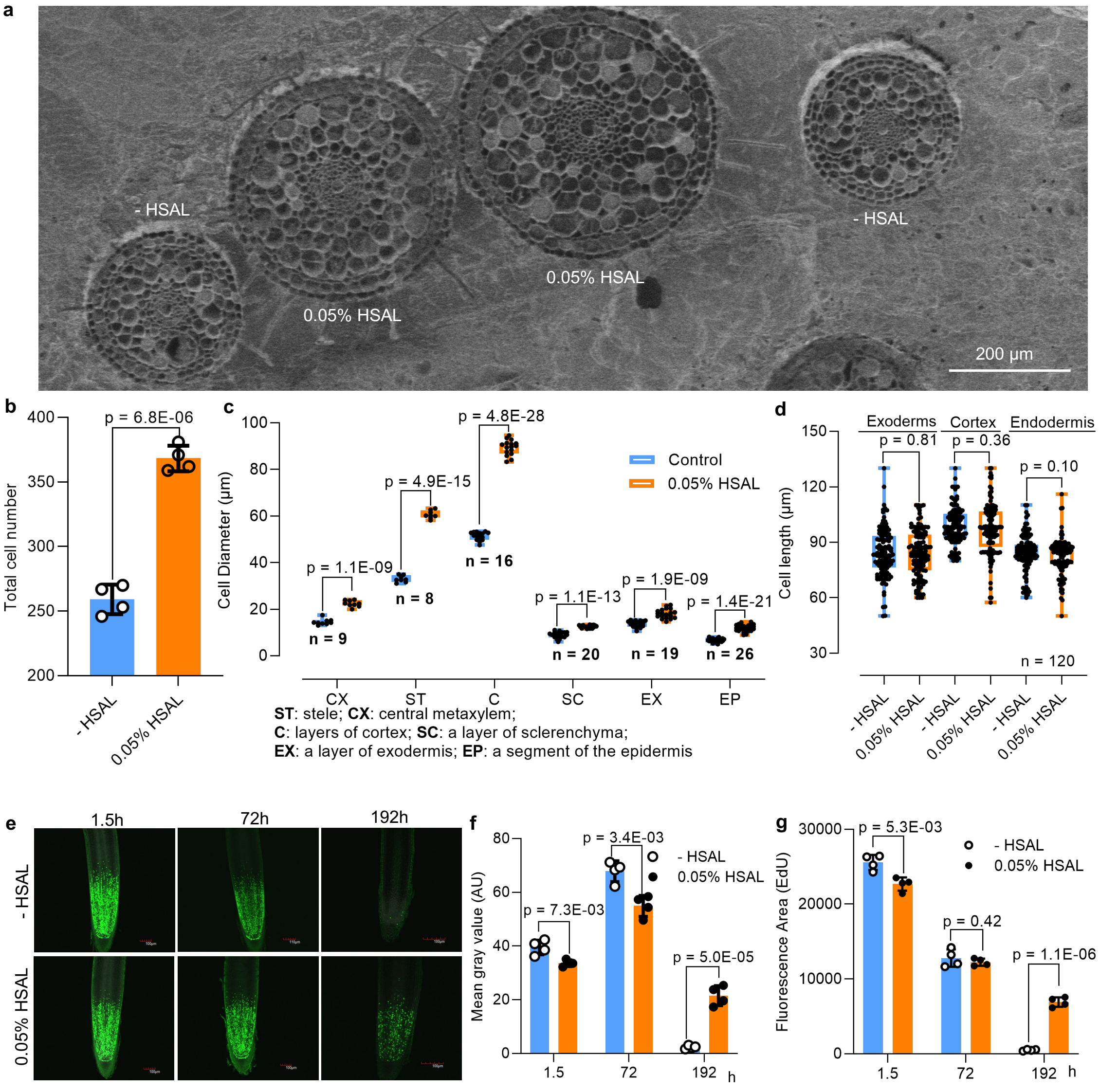


**Supplementary Fig. 5 | HSAL additive increases root diameter and cell activity of rice. a,** Cryo-SEM image showed the increase of 0.05% HSAL on the width of the differentiated rice (*Nippon bare*) roots for 7 days. **b,** The total cell number of different cell layers in the cross-sectional roots was shown as in (**a**). Graphs depict mean with standard deviation (error bars) and individual data points (n = 4 biologically independent replicates) **c,** Cell diameter for each cell layer shown in (**a**). **d,** The longitudinal cell length of the differentiated rice root. Boxes represent the 25$^{th}$–75$^{th}$ percentile range, white lines represent medians and whiskers show the minimum–maximum range. N = the number of cells in each cell layer in (**c**, **d**). **e,** Time-course EdU staining indicated the cell division activity in the root apex affected with 0.05% HSAL. **f, g,** The area (**f**) and intensity (**g**) of EdU fluorescence in the root meristems shown in (**e**). The graphs depicted the mean and standard deviation (error bars) and individual data points (n = 4 biologically independent replicates). Statistically significant differences between the means were analyzed with Student's *t*-test.

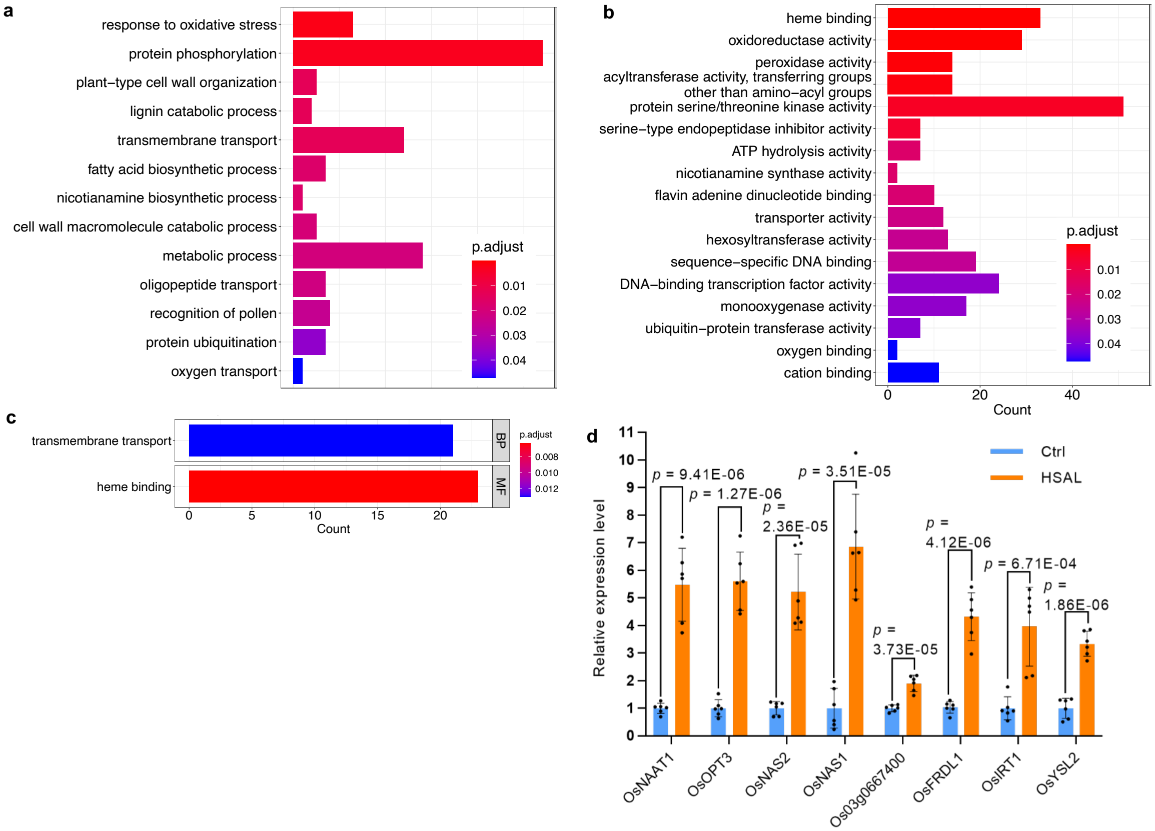


**Supplementary Fig. 6 | Gene Ontology (GO) term analysis of the differentially expressed genes regulated by HSAL.** The rice seedlings were grown in the hydroponic conditions for 7 days and treated with or without 0.05% HSAL for 12 h. Root tips of rice were collected for RNA-Seq analysis. DEGs were filtered at the threshold of False Discovery Rate (FDR) ≤ 0.05 and $Log_2$(Fold Change) ≥ 1 as shown in Fig. 4. (**a**, **b**) Enriched GO terms in Biological Process (**a**) and Molecular Function (**b**) categories of 956 upregulated DEGs. (**c**) Enriched Gene Ontology (GO) terms in Biological Process (BP) Molecular Function (MF) categories in 1112 downregulated DEGs. GO enrichment was carried out with the over representation analysis in (**a, b, c**). *p*-values were calculated by hypergeometric distribution multiple comparison followed by Benjamini-Hochberg adjustment. (**d**) HSAL additive increases the expression of iron transport-related genes in rice root tips. After grown in hydroponics for 7 days, the rice seedlings were treated with and without 0.05% HSAL for 12 h. The root tips were harvested to extract RNA for qRT-PCR. The graphs depicted the mean with standard deviation (error bars) and individual data points. *p*-value of statistical difference between the control and 0.05% HSAL was analyzed with Student's *t*-test (n = 6 biologically independent samples).

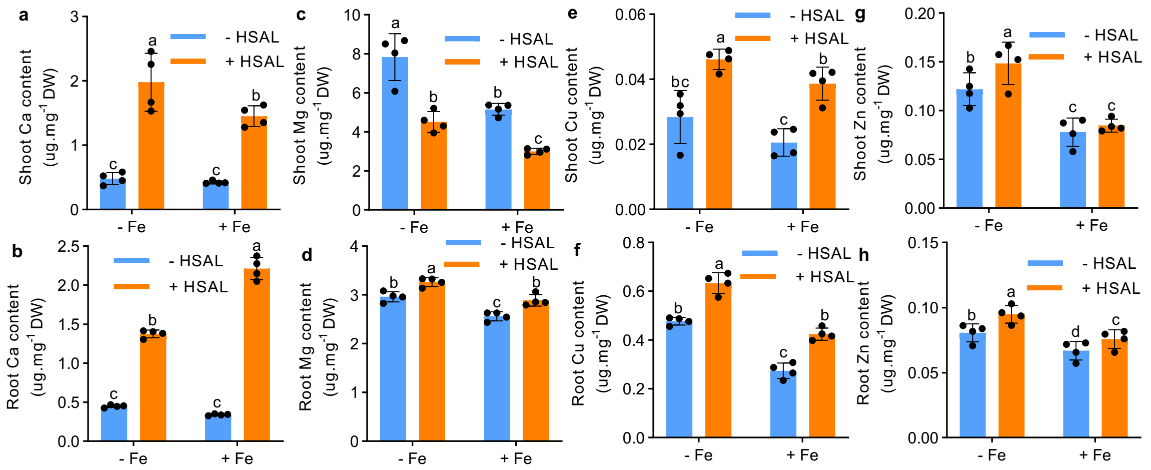


**Supplementary Fig. 7 | The regulation of HSAL additive on the nutrient content of rice seedlings**. The rice seedlings (*Nipponbare*) were grown under iron deficiency (without EDTA-Fe (II) addition) and iron sufficient (36 μM EDTA-Fe (II)), and treated with or without 0.05% HSAL for 10 days. Accumulation of Ca (**a**), Mg (**c**), Cu (**e**), Zn (**g**) in shoots and accumulation of Ca (**b**), Mg (**d**), Cu (**f**), and Zn (**h**) in roots. The graphs depicted the mean with standard deviation (error bars) and individual data points in (**a-h**). Different letters indicated the statistically significant differences among the means by two-way ANOVA analysis with Tukey's *HSD* test ($p < 0.05$, n = 4 biologically independent replicates).

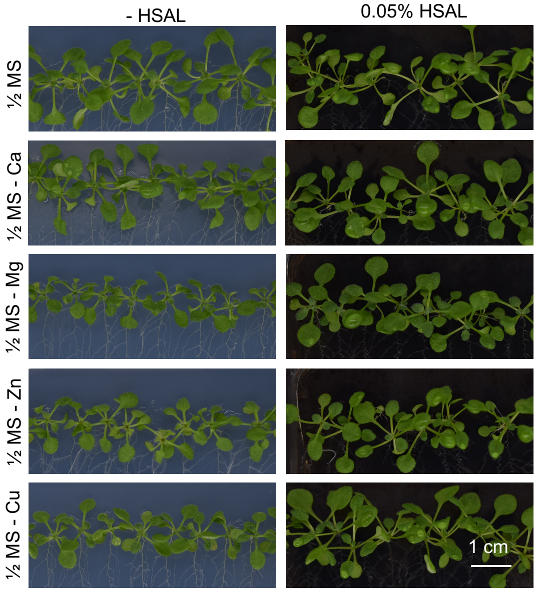


**Supplementary Fig. 8 | The recovery of HSAL additive on the growth defect of *Arabidopsis thaliana* caused by the deficiency of different nutrients.** The left panels showed *Arabidopsis thaliana* seedlings grown in the given nutrient conditions without added with HSAL (-HSAL), while right panels showing the seedlings grown in the corresponding nutrient conditions added with 0.05% HSAL. The panels from top to bottom showed *Arabidopsis* seedlings grown in the control nutrient condition (½ MS), calcium deficiency (½ MS - Ca); magnesium deficiency (½ MS - Mg); zinc deficiency (½ MS - Zn) and copper deficiency (½ MS - Cu), respectively. All the seedlings were grown for 10 days. The experiments have been independently repeated twice. The representative images were shown for the *Arabidopsis* leaf growth.

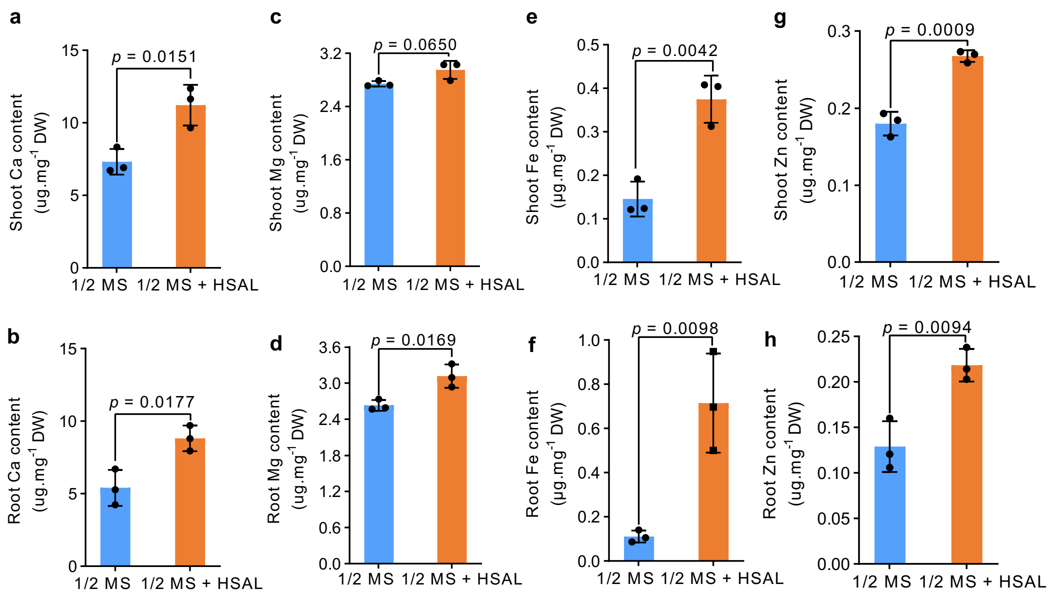


**Supplementary Fig. 9 | The regulation of HSAL additive on the nutrient content of *Arabidopsis* seedlings.** The seedlings were grown in the ½ MS medium (50 µM EDTA-Fe (II)) with or without 0.05% HSAL for 10 days. Accumulation of Ca (**a**), Mg (**c**), Fe (**e**), Zn (**g**) in shoots and accumulation of Ca (**b**), Mg (**d**), Fe (**f**), and Zn (**h**) in roots. The graphs depicted the mean with standard deviation (error bars) and individual data points in (**a-h**). Statistically significant differences between the means were analyzed with Student's t-test ($p < 0.05$, n = 3 biologically independent replicates).

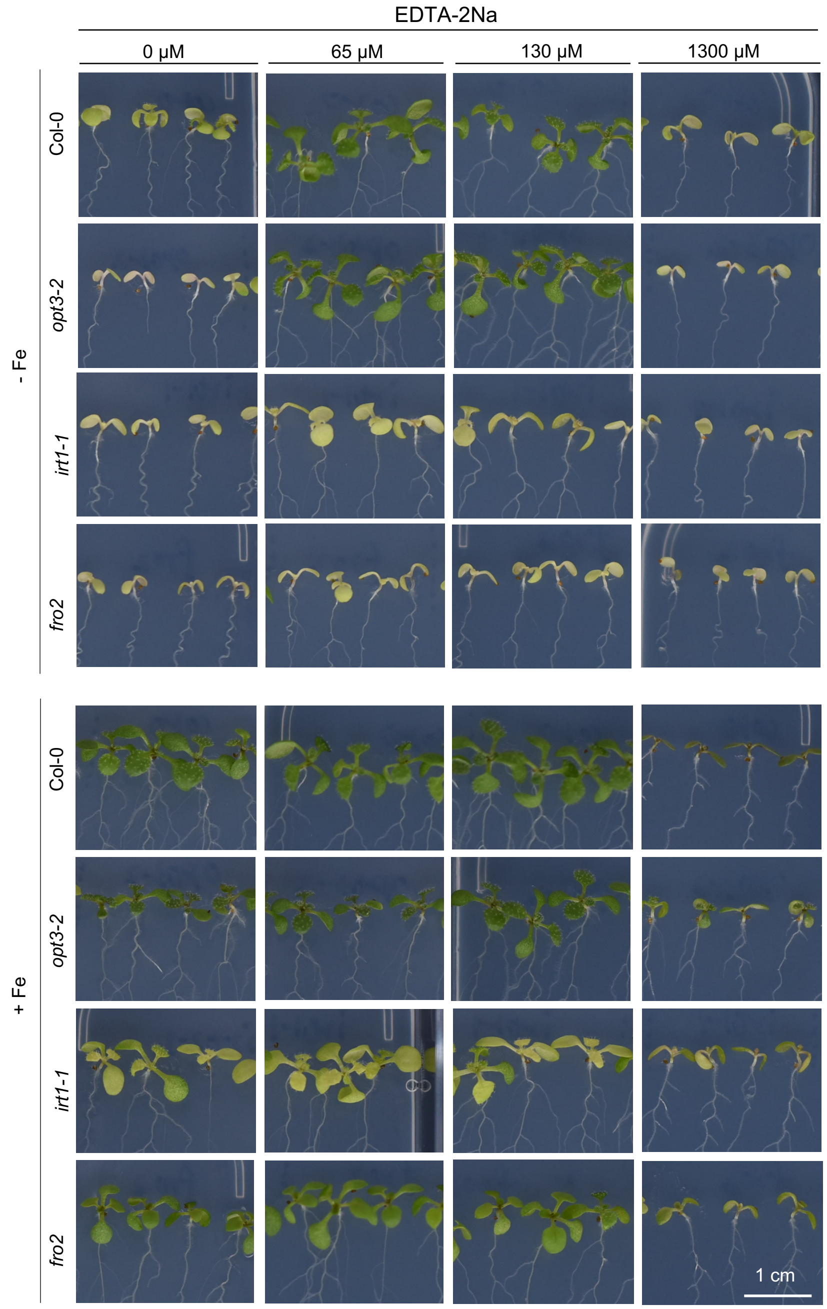


**Supplementary Fig. 10 | EDTA additive recovers the iron-deficiency phenotype of *Arabidopsis thaliana,* which depends on the FRO2/IRT1 iron transport pathway.** -Fe represented iron deficiency condition (½ MS without EDTA-Fe (III)), while + Fe indicated iron sufficient condition (½ MS with 50 μM EDTA-Fe (III)). Col-0 is wild-type *Arabidopsis*, while *opt3-2*, *irt1-1*and *fro2* represented three different iron transport defective *Arabidopsis* mutants. EDTA-$Na_2$ was supplied with four different concentrations (0, 65μM, 130μM and 1300μM) in the given ½MS growth medium. The seedlings have been grown for 9 days. The experiments have been independently repeated twice. The representative images were shown for *Arabidopsis* leaf growth.

**Supplementary Table 1. The Ca and Fe content in HSAL-metal complexes.**

| | Ca (μg. $g^{-1}$) | Fe (μg. $g^{-1}$) |
|---|---|---|
| HSAL | 70.86 ± 4.57 | 12.13 ± 2.55 |
| HSAL-$FeSO_4$ | 341.8 ± 88.11 | 6711 ± 640.0 |
| HSAL-$FeCl_3$ | 870.8 ± 93.50 | 2443 ± 289.7 |
| HSAL-$CaCl_2$ | 10047 ±974.7 | 54.27 ± 3.20 |

**Supplementary Table 2. Comparison of major nutrients among 0.05% HSAL, ½ MS and IRRI medium.**

| Elements | 0.05% HSAL (nM) | ½ MS (mM) | Ratio (0.05% HSAL/½ MS) | IRRI (mM) | Ratio (0.05%HSAL/IRRI) |
|---|---|---|---|---|---|
| K | 1.30 ± 0.53 | 10 | 0.00% | 1.00 | 0.00% |
| Ca | 162 ± 16.9 | 1.50 | 0.01% | 1.00 | 0.02% |
| Mg | 68.9 ± 7.77 | 0.75 | 0.01% | 1.70 | 0.00% |
| Fe | 30.5 ± 3.50 | 0.05 | 0.06% | 0.036 | 0.08% |
| Cu | 2.69 ± 0.31 | 0.00005 | 5.38% | 0.00016 | 1.68% |
| Zn | 1.01 ± 0.27 | 0.015 | 0.01% | 0.00015 | 0.67% |

Note: IRRI medium, Hydroponic Nutrient Solution Recipe based on the International Rice Research Institute.

**Supplementary Table 3. The increase of iron content in rice seedlings by 0.05% HSAL was not derived from HSAL-containing iron.**

| | Growth medium Fe ($\mu g. pot^{-1}$) | Root Fe ($\mu g. pot^{-1}$) | Shoot Fe ($\mu g. pot^{-1}$) | Total Fe ($\mu g. pot^{-1}$) |
|---|---|---|---|---|
| IRRI - Fe | 561.98 ± 49.67 | 10.67 ± 1.34 | 2.47 ± 0.35 | 13.07 ± 5.09 |
| IRRI-Fe+0.05% HSAL | 563.9 ± 49.48 | 36.03 ± 3.76 | 4.85 ± 0.33 | 40.83 ± 3.76 |
| Increase | 1.71 | 25.36 | 2.38 | 27.74 |

*Note*: The iron deficiency condition (IRRI - Fe) was IRRI medium without adding Fe-EDTA, while IRRI - Fe + 0.05% HSAL indicated the iron deficiency condition added with 0.05% HSAL. A pot contained 1 L hydroponics medium and grown with 12 rice seedlings.

**Supplementary Table 4. Increase of iron content in *Arabidopsis* seedlings by 0.05% HSAL under iron deficiency condition was not from HSAL-containing iron.**

| | Growth medium Fe (µg. plate$^{-1}$) | Root Fe (µg. plate$^{-1}$) | Shoot Fe (µg. plate$^{-1}$) | Total Fe (µg. plate$^{-1}$) |
|---|---|---|---|---|
| ½ MS - Fe | 31.96 ± 4.81 | 4.25 ± 0.46 | 14.70 ± 1.65 | 18.95 ± 1.87 |
| ½ MS - Fe + 0.05% HSAL | 32.04 ± 4.82 | 13.35 ± 3.98 | 20.20 ± 1.63 | 33.55 ± 3.87 |
| Increase | 0.085 | 9.1 | 5.5 | 14.6 |

*Note*: The iron deficiency condition (½ MS - Fe) was ½ MS salt removed of Fe-EDTA. ½ MS - Fe + 0.05% HSAL indicated the iron deficiency condition added with 0.05% HSAL. A plate contained 50 mL agar medium and grown with 15 *Arabidopsis* seedlings.
