# Supplementary material for: A lignin-derived material improves plant nutrient bioavailability and growth through its metal chelating capacity
Source: Nat Commun. 2023 Aug 11;14:4866. doi: 10.1038/s41467-023-40497-2 (PMC10421960; doi:10.1038/s41467-023-40497-2)
Supplement: Supplementary file 3 — Description of additional supplementary files [file 41467_2023_40497_MOESM3_ESM.pdf]

Description of Additional Supplementary Files

File name: **Supplementary Data 1**
Description: Differential expression genes (DEGs) were identified in the root tip of 7-day-old seedlings treated with or without HSAL for 12 h.

File name: **Supplementary Data 2**
Description: List of primer sequences for qRT-PCR in this Study.
